# The incidence of pulmonary thromboembolism in COVID-19 patients admitted to the intensive care unit: a meta-analysis and meta-regression of observational studies

**DOI:** 10.1186/s40560-021-00535-x

**Published:** 2021-02-22

**Authors:** Jun Jie Ng, Zhen Chang Liang, Andrew M. T. L. Choong

**Affiliations:** 1 Division of Vascular and Endovascular Surgery, Department of Cardiac, Thoracic and Vascular Surgery, National University Heart Centre, Level 9, NUHS Tower Block, 1E Kent Ridge Road, Singapore, 119228 Singapore; 2 Department of Surgery, Yong Loo Lin School of Medicine, National University of Singapore, Singapore, Singapore; 3 SingVaSC, Singapore Vascular Surgical Collaborative, Singapore, Singapore; 4 Department of Orthopaedic Surgery, National University Hospital, Singapore, Singapore

**Keywords:** Coronavirus disease 2019, COVID-19, Pulmonary thromboembolism, Intensive care unit, Critical care

## Abstract

**Objectives:**

Coronavirus disease 2019 (COVID-19) infection is associated with a prothrombotic state. We performed a meta-analysis of proportions to estimate the weighted average incidence of pulmonary thromboembolism (PTE) in COVID-19 patients who were admitted to the intensive care unit (ICU).

**Methods:**

We searched various medical databases for relevant studies from 31 December 2019 till 30 September 2020. We included observational studies that reported the incidence of PTE in COVID-19 patients admitted to the ICU. We extracted data related to study characteristics, patient demographics, and the incidence of PTE. Risk of bias was assessed by using the ROBINS-I tool. Statistical analysis was performed with R 3.6.3.

**Results:**

We included 14 studies with a total of 1182 patients in this study. Almost all patients in this meta-analysis received at least prophylactic anticoagulation. The weighted average incidence of PTE was 11.1% (95% CI 7.7% to 15.7%, *I*^2^ = 78%, Cochran’s *Q* test *P* < 0.01). We performed univariate and multivariate meta-regression, which identified the proportion of males as a significant source of heterogeneity (*P* = 0.03, 95% CI 0.00 to − 0.09)

**Conclusion:**

The weighted average incidence of PTE remains high even after prophylactic anticoagulation. PTE is a significant complication of COVID-19 especially in critically ill patients in the ICU.

**Supplementary Information:**

The online version contains supplementary material available at 10.1186/s40560-021-00535-x.

## Background

Since the declaration of a global pandemic by the World Health Organization on 11 March 2020, more than 25 million people have been diagnosed with coronavirus disease 2019 (COVID-19). Among them, over 800,000 people have died [1]. A few distinct themes have emerged as we gradually understand more about the pathophysiology and clinical manifestations of COVID-19 infection. One of the more apparent themes is the hypercoagulable, prothrombotic state that critically ill COVID-19 patients are susceptible to [2]. Early studies first reported autopsy findings of micro-thrombus within the pulmonary vasculature of deceased COVID-19 patients [3]. At the same time, other studies started reporting about abnormal coagulation parameters and elevated D-dimer levels in critically ill COVID-19 patients [4, 5]. On the frontline, physicians treating critically ill COVID-19 patients started noticing an increase in thromboembolic events and line thrombosis [6]. Cognizant of the thromboembolic phenomenon associated with COVID-19, several institutions have published observational studies that reported the incidence of thromboembolic events such as pulmonary thromboembolism (PTE). In this study, we aim to quantitatively synthesize available literature by using meta-analysis of proportions to estimate the weighted average incidence of PTE in critically ill COVID-19 patients admitted to the intensive care unit (ICU).

## Methods

### Study protocol

We conducted this systematic review and meta-analysis following the Cochrane Handbook for Systematic Reviews of Interventions and reported it in accordance with the Preferred Reporting Items for Systematic Reviews and Meta-analyses (PRISMA) guidelines [7, 8]. We formulated the study protocol for this systematic review and meta-analysis in an a priori fashion and published it in PROSPERO (CRD42020188647). Our study protocol is also available as Additional file 1.

### Search strategy

We formulated the search strategy after discussion and consensus by all authors. The search strategy included various combinations and permutations of the following search terms: “coronavirus,” “COVID-19,” “SARS-CoV-2,” “2019-nCoV,” “thrombus,” “thrombo*,” “embolus,” and “emboli*.” Our detailed search protocol is available as Additional file 2. We identified studies by conducting an exhaustive literature search using MEDLINE via PubMed, Embase, and Web of Science. We modified the search syntax for compatibility as required for each database. We only included studies published after 31 December 2019, which corresponds to the date when Chinese officials first reported a cluster of patients diagnosed with pneumonia of unknown cause in Wuhan, Hubei Province, to the World Health Organization [9]. We did not restrict language for the search. After eligible full-text studies were identified, we performed manual backward reference searching to ensure all relevant studies were included. We only included studies that were published in a peer-reviewed journal. We performed a repeat search on 30 September 2020 before submission to ensure no studies were missed.

### Eligibility criteria

We included prospective and retrospective observational studies that reported the incidence of PTE in COVID-19 patients admitted to the ICU for treatment. We excluded individual case reports or case series on PTE in COVID-19 patients. We excluded studies that determined the incidence of PTE by reviewing cross-sectional chest imaging regardless of clinical indication. Including these studies may over-estimate the number of patients with PTE as many of these patients with incidental imaging findings of PTE may be clinically asymptomatic. We also excluded studies that had reported the incidence of venous thromboembolism in general without reporting the specific incidence of PTE. Lastly, we excluded studies published in pre-print servers as they are not peer-reviewed and might be prone to bias.

### Selection of studies and data extraction

We imported the search items into a commercially available reference manager for deduplication. Following deduplication, two authors (JN and ZL) screened the titles and abstracts for relevant studies. After screening, and obtaining the full-text manuscript of relevant studies, the same two authors reviewed them carefully for inclusion into our systematic review and meta-analysis. Disagreements during abstract and title screening or full-text review were resolved by consensus after discussion with a third author (AC). An author (JN) extracted relevant data from the included studies, and another author (AC) verified the accuracy of the extracted data. We extracted the following variables from the included studies: study first author, study location, study period, study type, study population, study sample size, demographical information (age, gender, body-mass index), comorbidities (diabetes mellitus, hypertension, active malignancy, previous venous thromboembolism), laboratory parameters on admission to ICU (platelet count, D-dimer levels), venous thromboembolism prophylaxis regimens, the proportion of patients on prophylactic or therapeutic anticoagulation, indication for performing PTE imaging, the incidence of PTE, and follow-up period.

### Study outcome

The primary outcome is to estimate and report the weighted average incidence of PTE in critically ill COVID-19 patients admitted to the ICU. We considered a positive diagnosis of PTE only if the diagnosis was confirmed by contrast-enhanced computed tomographic imaging of the chest. The secondary outcome is to assess for moderators that could potentially affect the primary outcome.

### Risk of bias assessment

Two authors (JN and ZL) assessed the risk of bias of all included studies using the ROBINS-I tool [10]. Disagreements were resolved by consensus after discussion with a third author (AC). We utilized the ROBINS-I tool as the studies included in the meta-analysis are non-randomized observational studies. The ROBINS-I tool was explicitly designed to assess the risk of bias in non-randomized studies in seven domains—bias due to confounding, selection bias, bias in classification of interventions, bias due to deviations from intended interventions, bias due to missing data, bias in the measurement of outcomes, and bias in the selection of reported results. Each domain will be graded to be either at low risk, moderate risk, or high risk of bias. For a domain to be graded low risk, the domain must be comparable to a well-performed randomized trial. For a domain to be graded moderate risk, the domain must be sound for a non-randomized study but cannot be comparable to a well-performed randomized trial. For a domain to be graded serious risk, it means that the domain has some critical issues that need to be addressed. Each included study would be appraised based on each domain’s summative grading to deduce the overall risk of bias. If all the domains within a study were graded to have a low risk of bias, the study’s overall risk of bias would be low. If any of the domains within a study were graded to have a moderate risk of bias, the study’s overall risk of bias would be moderate. If any of the domains within a study were graded to have a serious risk of bias, the study’s overall risk of bias would be serious.

### Data analysis

We performed statistical analysis using the meta and metafor packages with R 3.6.3 (R Foundation for Statistical Computing, Vienna, Austria). A frequentist approach was utilized. A meta-analysis of proportions was performed using a random-effects model (DerSimonian and Laird) with logit transformation of observed proportions. The primary outcome was reported as proportions with their respective 95% confidence intervals (CI). We assessed statistical heterogeneity using the Cochran’s *Q* test and *I*^2^ statistic. For Cochran’s *Q* test, we used a *P* value of less than 0.1 to represent significant heterogeneity of intervention effects. For the *I*^2^ statistic, a value of more than 50% represented substantial statistical heterogeneity. Initially, as part of our study protocol, we planned to perform further sensitivity analyses such as leave-one-out analysis to explore the sources of study heterogeneity. However, we eventually refrained from performing sensitivity analyses as meta-regression analysis was sufficient to account for the possible moderators that might contribute to statistical heterogeneity. We converted median and interquartile range values to mean and standard deviation for meta-regression analysis using a validated method [11]. We evaluated publication bias with a funnel plot and rank correlation test.

## Results

### Study selection

**Fig. 1:**
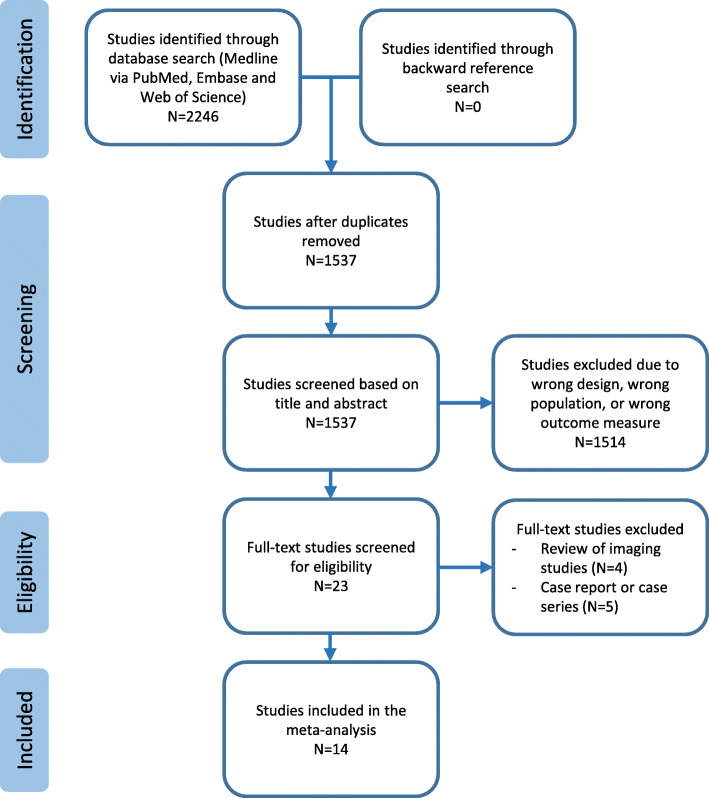
PRISMA flowchart for study selection

A thorough and systematic search was conducted according to the pre-defined search protocol specified in the methods section of this manuscript (Fig. 1). The search yielded a total of 2246 studies, of which 1537 studies remained after deduplication. Following title and abstract screening, we identified 23 studies for full-text review. After completion of the full-text review, we included 14 studies into this systematic review and meta-analysis [12–25].

### Risk of bias assessment

Risk of bias was assessed by using the ROBINS-I tool (Table 1) [10]. A single study was assessed to have a low risk of bias across all domains, and hence deemed to have a low overall risk of bias [14]. Nine studies were considered to have a moderate overall risk of bias, as one or more domains were deemed to be at moderate risk [13, 16, 17, 19–22, 24, 25]. Four studies were considered to have a serious overall risk of bias due to the presence of missing data such as patient comorbidities and ICU characteristics [12, 15, 18, 23].

**Table 1:**
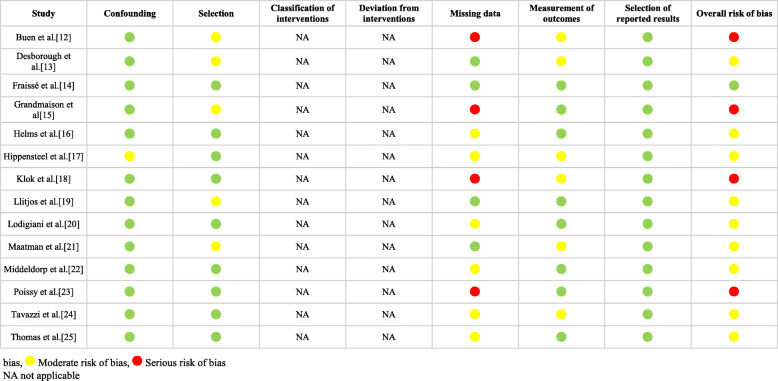
Risk of bias assessment with the ROBINS-I tool

Low risk of bias,  Moderate risk of bias,  Serious risk of bias

*NA* not applicable

### Characteristics of included studies

We included 14 studies with a total of 1182 patients into this systematic review and meta-analysis [12–25]. A summary of study characteristics can be seen in Table 2, whereas a summary of patient characteristics can be seen in Table 3. Four studies were conducted in France [14, 16, 19, 23], three in the Netherlands [12, 18, 22], two in Italy [20, 24], two in the UK [13, 25], two in the USA [17, 21], and one in Switzerland [15]. All studies were conducted between February 2020 and April 2020. Only five studies had reported the duration of follow-up, which varied from 7 to 28 days [13, 16, 18, 22, 25].

**Table 2:** Summary of study characteristics

Study	Study location	Study period	Indication for ICU admission	***N*** (ICU)	Prophylactic anticoagulation agent	Patients receiving therapeutic anticoagulation on ICU admission (%)	Patients receiving prophylactic anticoagulation on ICU admission (%)	Patients receiving at least anticoagulation on ICU admission (%)	Imaging Modality for PTE diagnosis	Indication for PTE imaging	Incidence of PTE (%)	Follow-up (days)
Beun et al. [12]	Netherlands	16 March–9 April	NR	75	NR	NR	NR	NR	CT scan	NR	26.7	NR
Desborough et al. [13]	UK	1 March–31 March	NR	66	Dalteparin	16.7	83.3	100	CT scan	NR	7.6	28
Fraissé et al. [14]	France	6 March–22 April	Respiratory failure	92	NR	53.3	46.7	100	CT scan	Clinical suspicion	20.7	NR
Grandmaison et al. [15]	Switzerland	NR	NR	29	Enoxaparin, UFH	3.4	89.7	93.1	CT scan	Clinical suspicion	6.9	NR
Helms et al. [16]	France	3 March–31 March	Acute respiratory distress syndrome based on Berlin definition	150	LMWH, UFH	30	70	100	CT scan	Worsening PaO_2_/FiO_2_ ratio, haemodynamic instability, dilated right ventricle, rapid increase in D-dimer	16.7	≥ 7
Hippensteel et al. [17]	USA	18 March–14 April	NR	91	NR	NR	NR	NR	CT scan	NR	5.5	NR
Klok et al. [18]	Netherlands	7 March–5 April	NR	184	Nadroparin	9.2	90.8	100	CT scan	NR	13.6	7 (1–13)
Llitjos et al. [19]	France	19 March–11 April	Respiratory failure	26	LMWH, UFH	69.2	30.8	100	CT scan	Persistent respiratory failure	23.1	NR
Lodigiani et al. [20]	Italy	13 February–10 April	NR	61	LMWH	3.3	96.7	100	CT scan	Worsening PaO_2_/FiO_2_ ratio, rapid increase in D-dimer	3.3	NR
Maatman et al. [21]	USA	12 March–31 March	SpO_2_ ≤ 94%, RR ≥ 30, PaO_2_/FiO_2_ ratio ≤ 300 mmHg, or requiring mechanical ventilation	109	Enoxaparin, UFH	6.4	93.6	100	CT scan	NR	4.6	NR
Middeldorp et al. [22]	Netherlands	2 March–12 April	NR	75	Nadroparin	9.3	90.7	100	CT scan	Worsening hypoxaemia	14.7	15 (9–20)
Poissy et al. [23]	France	27 February–31 March	NR	107	LMWH, UFH	NR	NR	NR	CT scan	Acute deterioration of haemodynamic and respiratory status	20.6	NR
Tavazzi et al. [24]	Italy	NR	NR	54	LMWH	0	100	100	CT scan	NR	3.7	NR
Thomas et al. [25]	UK	15 March–14 April	NR	63	Dalteparin	0	100	100	CT scan	Unexplained hypotension or hypoxia disproportionate to pneumonia	7.9	8 (1–28)

*ICU* intensive care unit, *BMI* body-mass index, *PTE* pulmonary thromboembolism, *CT* computed tomographic, *NR* not reported, *LMWH* low-molecular-weight heparin, *UFH* unfractionated heparin, *RR* respiratory rate

**Table 3:** Summary of patient characteristics

Study	Age (years)	Male (%)	BMI (kg/m^**2**^)	DM (%)	Hypertension (%)	Malignancy (%)	Previous VTE (%)	Platelet count (× 10^**9**^/L)	D-dimer (mg/L)	Patients intubated (%)	Patients on inotropes (%)	Patients on RRT (%)	Patients on ECMO (%)
Beun et al. [12]	NR	NR	NR	NR	NR	NR	NR	NR	NR	NR	NR	NR	NR
Desborough et al. [13]	59 (49–66)	72.7	28 (24–34)	40.9	45.5	7.6	7.6	207 (154–272)	2.4 (1.1–6.2)	78.8	47	27.3	12.1
Fraissé et al. [14]	61 (55–70)	79.3	30 (26–35)	38	64.1	NR	5.4	227 (182–307)	2.4 (1.7–7.9)	89.1	62	23.9	NR
Grandmaison et al. [15]	NR	72.1	NR	NR	NR	6.9	6.9	NR	NR	NR	NR	NR	NR
Helms et al. [16]	63 (53–71)	81.3	NR	20	NR	6	5.3	200 (152–267)	2.3 (1.2–20)	100	NR	NR	8
Hippensteel et al. [17]	56 ± 16	58.2	32.4 ± 9.9	30.8	NR	3.3	NR	200 ± 91	3.0 ± 10.3	84.6	67	NR	NR
Klok et al. [18]	64 ± 12	75.5	NR	NR	NR	2.7	NR	NR	NR	NR	NR	12.5	NR
Llitjos et al. [19]	68 (52–75)	76.9	30.2 (25.5–33.5)	NR	84.6	0	3.8	234 (169–306)	1.8 (1.1–2.9)	100	88.5	15.4	7.7
Lodigiani et al. [20]	61 (55–69)	80.3	NR	18	42.6	3.3	0	NR	NR	NR	NR	NR	NR
Maatman et al. [21]	61 ± 16	56.9	34.8 ± 11.8	39.4	67.9	NR	NR	207 (152–255)	0.5 (0.3–1.0)	94.5	64.2	14.7	2.8
Middeldorp et al. [22]	62 ± 10	77.3	27 (24–29)	NR	NR	4	2.7	251 ± 89	2 (0.8–8.1)	NR	NR	NR	NR
Poissy et al. [23]	NR	NR	NR	NR	NR	NR	NR	NR	NR	NR	NR	NR	NR
Tavazzi et al. [24]	68 ± 7	83.3	29.3 ± 4.4	NR	NR	NR	NR	NR	NR	100	NR	NR	NR
Thomas et al. [25]	NR	69.8	NR	NR	NR	1.6	1.6	NR	3.9^a^ (1.2–36.3)	82.5	NR	36.5	NR

*BMI* body-mass index, *DM* diabetes mellitus, *CKD* chronic kidney disease, *VTE* venous thromboembolism, *RRT* renal replacement therapy, *ECMO* extracorporeal membrane oxygenation, *NR* not reported

Unless otherwise stated, all values are represented in percentages (%), mean ± standard deviation, or median (interquartile range)

^a^Value represented as median (range)

### Indication for ICU admission

Only four studies had reported their indication for ICU admission [14, 16, 19, 21]. Two studies defined their ICU admission criteria as any patient with respiratory failure [14, 19]. Helms et al. defined their ICU admission criteria as patients who have acute respiratory distress syndrome based on the Berlin 2012 definition [16, 26], whereas the study by Maatman et al. defined their ICU admission criteria as any patient with an oxygen saturation of 94% or less, respiratory rate of 30 breaths per minute or more, PaO_2_/FiO_2_ ratio of 300 mmHg or less, or if requiring mechanical ventilation [21].

### Prophylactic anticoagulation regimen and compliance

Eleven studies reported using either low-molecular-weight heparin (enoxaparin, nadroparin, dalteparin, or unspecified) or unfractionated heparin for venous thromboembolism prophylaxis in varying doses [13, 15, 16, 18–25]. The majority of studies had also reported information on the proportion of patients receiving therapeutic or prophylactic anticoagulation in ICU. The proportion of patients started on therapeutic anticoagulation in ICU varied from 0 to 69.2%, while the proportion of patients started on prophylactic anticoagulation varied from 30.8 to 100%. Overall, in 10 out of the 11 studies with sufficient information on anticoagulation practices, 100% of patients received at least prophylactic anticoagulation [13, 14, 16, 18–22, 24, 25]. In the study by Grandmaison et al., 93.1% of patients received at least prophylactic anticoagulation [15].

### Modality and indication for pulmonary thromboembolism imaging

Contrast-enhanced computed tomographic scan was the principal modality used to diagnose PTE in all included studies [12–25]. Eight studies specifically reported the indication for performing PTE imaging [14–16, 19, 20, 22, 23, 25]. All the eight studies adopted a selective approach based on the patient’s clinical condition to decide if PTE imaging was required. In these studies, PTE imaging was only performed if there was a clinical suspicion of PTE, such as if patients had persistent respiratory failure, deteriorating respiratory function or hemodynamic status, or a rapid increase in D-dimer levels.

### Primary outcome: incidence of pulmonary thromboembolism

**Fig. 2:**
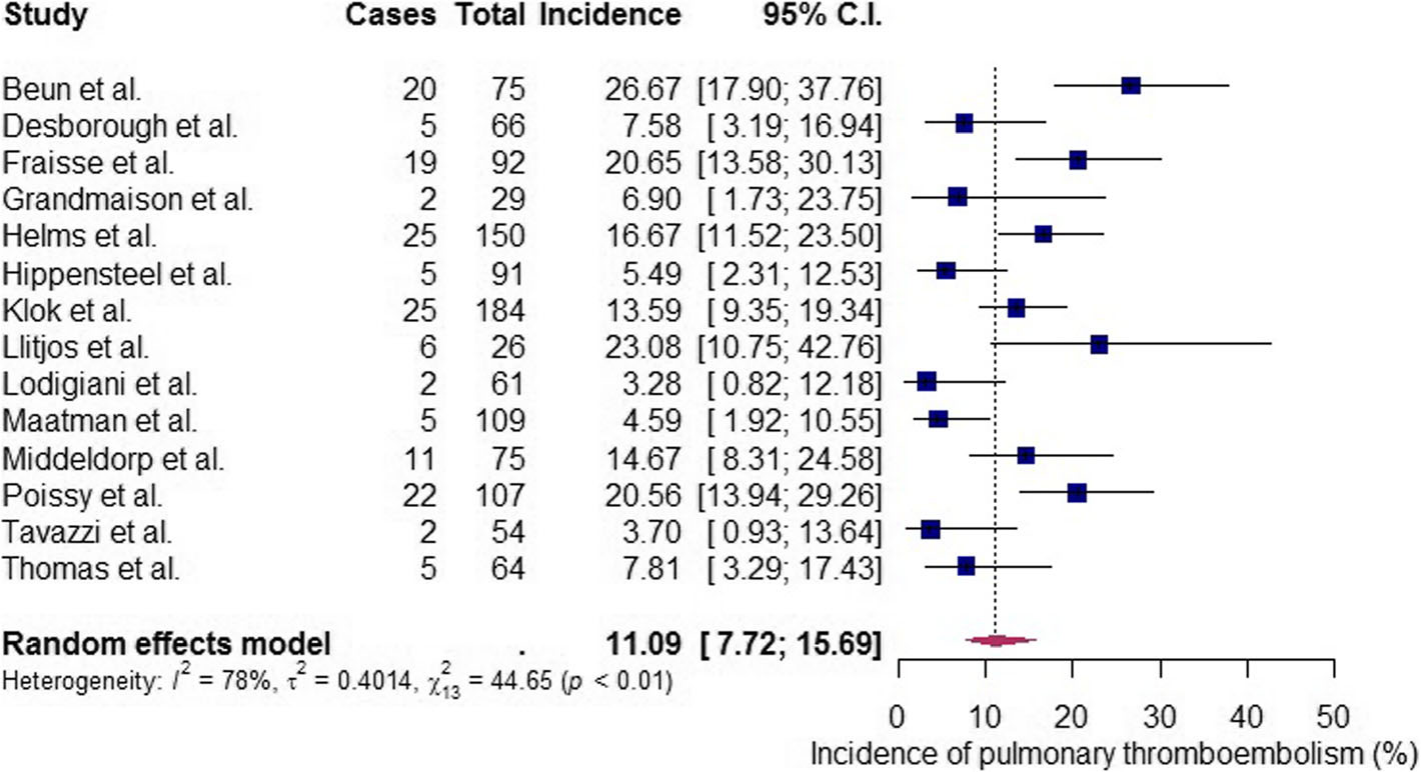
Forest plot showing the weighted average incidence of pulmonary thromboembolism in critically ill COVID-19 patients admitted to the intensive care unit for treatment

The reported incidence of PTE ranged from 3.3 to 26.7%. Including all the 14 studies, the weighted average incidence of PTE in COVID-19 patients after admission to the intensive care unit was 11.1% (95% CI 7.7 to 15.7%, *I*^2^ = 78%, Cochran’s *Q* test *P* < 0.01) after a random-effects meta-analysis of proportions (Fig. 2) [12–25]. Significant statistical heterogeneity was present as evidenced by high *I*^2^ value and a Cochran’s *Q* test *P* value of less than 0.1.

### Meta-regression and moderator assessment

Meta-regression with a mixed-effects model was performed to examine if the observed heterogeneity could be contributed by possible moderators such as patient or study characteristics (Table 4). Univariate meta-regression revealed that the proportion of male patients, platelet count on admission to ICU, and proportion of patients on therapeutic anticoagulation were possible significant moderators. These three significant moderators were added into the multivariable meta-regression model for further analysis. Multivariable meta-regression revealed that the proportion of male patients remained as the only significant moderator in this meta-analysis. A higher proportion of males were associated with a higher incidence of PTE.

**Table 4:** Meta-regression analysis

	Univariate analysis				Multivariate analysis			
Variables	Coeff	SE	95% CI	***P***	Coeff	SE	95% CI	***P***
Sample size	0.00	0.00	− 0.01–0.01	0.55	–	–	–	–
Age (years)	0.06	0.08	− 0.09–0.22	0.40	–	–	–	–
Male gender (%)	0.06	0.02	0.03–0.09	< 0.01	0.05	0.02	0.00–0.09	0.03
Body-mass index (kg/m^2^)	− 0.14	0.12	− 0.37–0.10	0.25	–	–	–	–
Diabetes mellitus (%)	0.00	0.04	− 0.08–0.08	0.95	–	–	–	–
Hypertension (%)	0.04	0.03	− 0.01–0.09	0.15	–	–	–	–
Active malignancy (%)	− 0.06	0.09	− 0.24–0.13	0.54	−	–	–	–
Previous VTE (%)	0.08	0.11	− 0.13–0.28	0.48	–	–	–	–
Platelet count	0.03	0.01	0.01–0.05	0.01	0.01	0.01	− 0.01–0.02	0.42
D-dimer level	0.00	0.00	− 0.00–0.01	0.44	–	–	–	–
Patients on therapeutic anticoagulation (%)	0.02	0.01	0.01–0.03	< 0.01	0.01	0.01	− 0.01–0.03	0.24
Patients intubated (%)	0.03	0.04	− 0.04–0.10	0.43	–	–	–	–
Patients on inotropes (%)	0.03	0.03	− 0.03–0.09	0.36	–	–	–	–
Patients on RRT (%)	− 0.02	0.04	− 0.09–0.06	0.66	–	–	–	–
Patients on ECMO (%)	0.07	0.15	− 0.21–0.35	0.63	–	–	–	–

*BMI* body mass index, *Coeff* coefficient, *CI* confidence interval, *ECMO* extracorporeal membrane oxygenation, *RRT* renal replacement therapy, *SE* standard error, *VTE* venous thromboembolism

### Publication bias

**Fig. 3:**
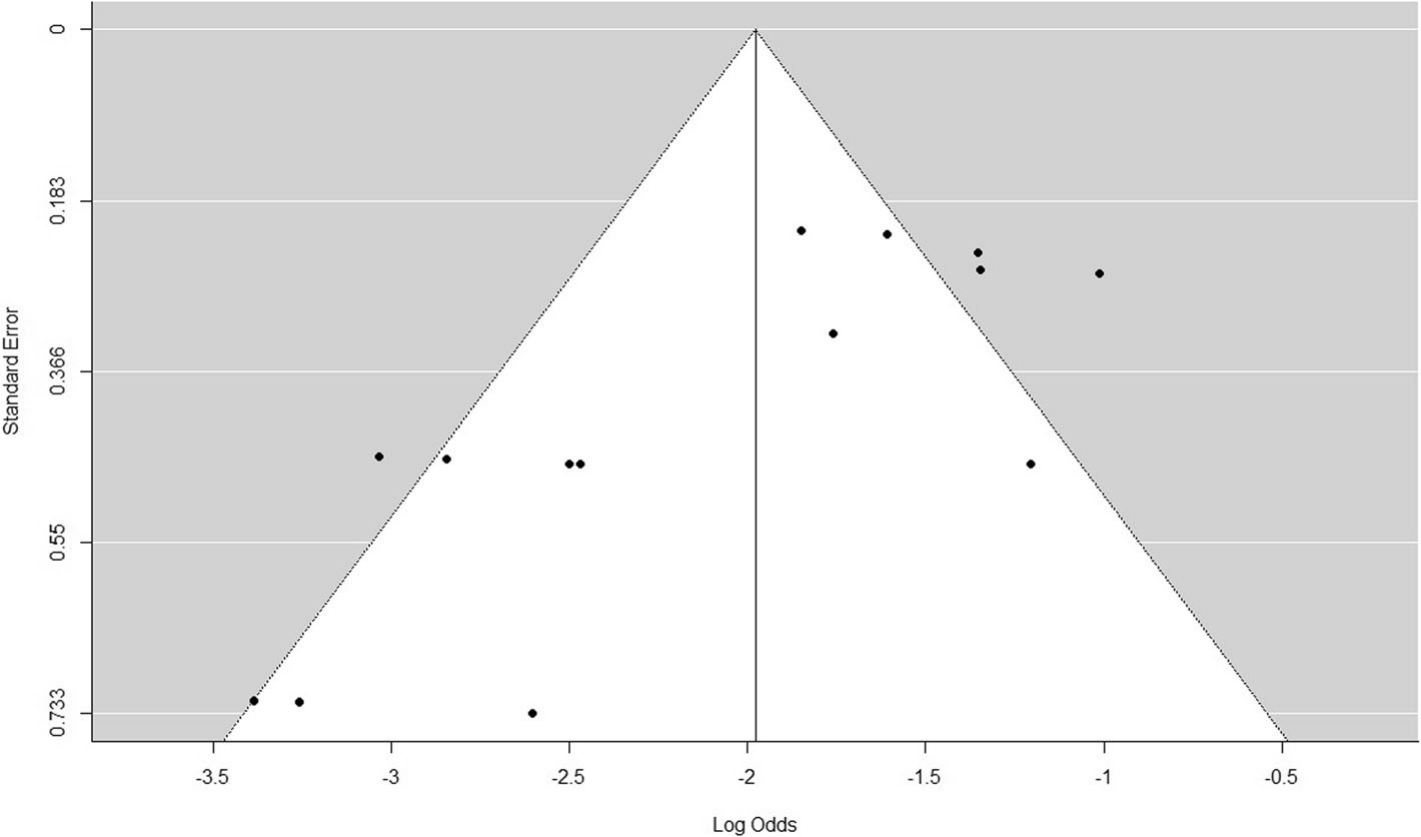
Funnel plot for assessment of publication bias

We assessed publication bias by using a funnel plot and the rank correlation test. The funnel plot of all included studies is as shown in Fig. 3. The rank correlation test proved that there was no significant funnel plot asymmetry (*P* = 0.19).

## Discussion

In this meta-analysis, we found that the weighted average incidence of PTE in critically ill COVID-19 patients after admission to the ICU to be 11.1% (95% CI 7.7 to 15.7%). Although other similar meta-analyses have been performed and published in the literature, our study is unique due to several reasons [27]. Firstly, this study was performed specifically to evaluate the risk of pulmonary thromboembolism in COVID-19 patients admitted to the ICU instead of a general hospitalized population. Second, we had strict inclusion criteria and only included studies that started with a cohort of ICU patients. We excluded studies that had identified patients with PTE by examining the reports of all COVID-19 patients who had undergone a computed tomographic scan of the chest. Inclusion of these studies might overestimate the true incidence of symptomatic PTE. Lastly, our study is the only meta-analysis that had included a meta-regression analysis to identify and examine the impact of confounder variables on the study effect size. The results from this meta-analysis can be used for several important purposes. First, it can aid in the planning of healthcare resources. As the number of COVID-19 patients continues to rise, more patients will be admitted to ICU and subsequently diagnosed with PTE. The weighted average incidence of PTE could be used to forecast the need for valuable healthcare resources such as mechanical ventilators as COVID-19 patients with PTE have been shown to require a longer duration of mechanical ventilation [28]. Next, the results from this meta-analysis confirm the thromboembolic risk associated with COVID-19 infection and that a diagnosis of PTE should be considered in any patient with respiratory deterioration. At the same time, patient and family members can be educated on possible complications such as PTE that can arise if admission to ICU is required. Lastly, results from our meta-analysis can also be used to justify the conduct of clinical trials that aim to reduce the risk of thromboembolic events in COVID-19 patients.

We identified the proportion of males to be a significant moderator and significant source of statistical heterogeneity in the incidence of PTE after univariate and multivariate meta-regression analysis. Our analysis shows that studies with a higher proportion of male patients had a higher incidence of reported PTE. This observed phenomenon can be corroborated by several studies performed in the past that have demonstrated a higher risk of first episode or recurrent venous thromboembolism in males than females [29]. Several hypotheses such as genetic variations or differences in environmental factors have been suggested to account for the differential risks of venous thromboembolism, but the evidence remains unclear [30].

The significant statistical heterogeneity of our primary outcome could also be explained by other moderators that were not included in our meta-regression analysis. One possibility that cannot be easily analyzed or accounted for would be how study location and study period can affect the incidence of PTE. Studies by Lodigiani et al. and Tavazzi et al., which were both conducted in Italy, had reported relatively low PTE incidences of 3.3% and 3.7% respectively compared to studies conducted outside of Italy [20, 24]. We postulate several possible reasons for the observed geographical disparity in reported PTE incidences. First, Lodigiani et al. had reported that only 13.1% of their study population had PE imaging performed [20]. Compared to other studies included in this meta-analysis, this seemed to be considerably lower. As such, PTE could have been underdiagnosed. On the contrary, other studies with more widespread PTE imaging may have diagnosed many patients with subclinical or asymptomatic PTE. Several retrospective studies reviewed CT scans performed COVID-19 patients regardless of clinical context had reported radiological findings of PTE in up to 50% of patients [28, 31]. Undoubtedly, most PTE findings in these radiological studies may be clinically insignificant. Furthermore, resource constraints secondary to the COVID-19 pandemic might contribute to the possible geographical disparity in PTE incidence. The studies by Lodigiani et al. and Tavazzi et al. were conducted in the Lombardy region of Italy, which had the most number of COVID-19 cases and the highest case fatality rate in Italy [32]. As such, the institutions at which the studies were conducted might have faced possible resource constraints that may have inevitably led to a more selective or conservative approach to PTE imaging and diagnosis [33]. Lastly, other moderators such as follow-up duration might have also contributed to the statistical heterogeneity, but cannot be adequately evaluated due to missing data.

Our study also provides some insight into the various prophylactic anticoagulation regimens adopted by different institutions. Interestingly, two studies conducted in the Netherlands had doubled the doses of their anticoagulation regimens around late March and early April [18, 22]. This is most likely due to an increased awareness of the thromboembolic manifestations of COVID-19. Ten studies had also reported that 100% of their study population had received at least prophylactic anticoagulation [13, 14, 16, 18–22, 24, 25]. However, despite a high degree of compliance to prophylactic anticoagulation, a considerable proportion of critically ill COVID-19 patients still developed PTE. Several studies have suggested an intermediate dose or therapeutic anticoagulation regimen for this group of patients due to this phenomenon [34, 35]. Several institutions worldwide are currently planning or have begun recruiting for clinical trials to evaluate the effects of higher dose anticoagulation for critically ill COVID-19 patients [36].

There are several limitations to our meta-analysis. First, we could not fully account for the differences in study population and study design. Many of the included studies did not report essential information such as the indication for ICU admission, exact type and dosages of anticoagulation therapy used, disease severity, and use of adjunctive therapies such as rehabilitation and follow-up duration. Due to significant missing data, 4 out of the 14 included studies were deemed to have high overall risk of bias. Next, we were unable to assess the effect of anticoagulation use on the incidence of clinically relevant adverse events such as minor or major hemorrhage as the included studies did not report them. Such information is vital as clinicians routinely need to decide between anticoagulation use and risk of hemorrhage. Lastly, we were also unable to assess the effect of PTE incidence on mortality, as most studies did not report mortality outcomes.

## Conclusion

In conclusion, the weighted average incidence of PTE in critically ill COVID-19 patients admitted to the ICU for treatment was 11.1%, even though almost all patients received at least prophylactic doses of anticoagulation therapy. Although there was significant heterogeneity in our meta-analysis, we identified the proportion of male patients as a significant moderator and contributor of heterogeneity. Most included studies have moderate to serious risks of bias, and results should be interpreted with caution. Clinicians should be aware that PTE can occur in a significant proportion of COVID-19 patients receiving ICU care despite adequate prophylactic anticoagulation and should investigate further should clinical suspicion arises. Moving ahead, more studies are also needed to determine the optimal anticoagulation strategy in critically ill COVID-19 patients.

## Supplementary Information

**Additional file 1.** Study protocol.

**Additional file 2.** Search strategy and keywords.

## Data Availability

The datasets used and/or analyzed during the current study are available from the corresponding author on reasonable request.

## Abbreviations

COVID-19: Coronavirus disease 2019
PTE: Pulmonary thromboembolism
ICU: Intensive care unit
CI: Confidence intervals
