# Supplementary material for: The incidence of pulmonary thromboembolism in COVID-19 patients admitted to the intensive care unit: a meta-analysis and meta-regression of observational studies
Source: J Intensive Care. 2021 Feb 22;9:20. doi: 10.1186/s40560-021-00535-x (PMC7897892; doi:10.1186/s40560-021-00535-x)
Supplement: Supplementary file 1 — Additional file 1. Study protocol. [file 40560_2021_535_MOESM1_ESM.docx]

**Study protocol**

1. Review question – What is the average weighted incidence of pulmonary thromboembolism in COVID-19 patients treated in the intensive care unit?
2. Search strategy – Refer to additional file 2 for our detailed search strategy.
3. Determine eligibility criteria
   1. Prospective or observational retrospective studies
   2. Reported incidence of pulmonary thromboembolism in COVID-19 patients in intensive care unit setting
   3. Pulmonary thromboembolism must be proven on computed tomographic scan
   4. Studies must start with cohort of COVID-19 intensive care unit patients and not cohort of patients who undergone computed tomographic scan of the chest
   5. Peer-reviewed articles
4. Determine primary outcome of study – weighted average incidence of pulmonary thromboembolism in COVID-19 patients that were admitted to the intensive care unit
5. Perform search in PubMed, Embase and Web of Science.
6. Selection of studies based on eligibility criteria
7. Data extraction
8. Risk of bias assessment with Robins-I tool
9. Qualitative assessment of studies
10. Quantitative assessment of studies ascertain primary outcome
11. Sensitivity analysis with Baujat plot and leave-one-out analysis
12. Meta-regression to assess possible sources of statistical heterogeneity with predefined covariates of sample size, age, gender, body-mass index, diabetes mellitus, hypertension, active malignancy, previous venous thromboembolism, platelet count, d-dimer level, patients on therapeutic anticoagulation, patients intubated, patients on intropes, patients on renal replacement therapy and patients on extracorporeal membrane oxygenation.
