# Supplementary material for: The incidence of pulmonary thromboembolism in COVID-19 patients admitted to the intensive care unit: a meta-analysis and meta-regression of observational studies
Source: J Intensive Care. 2021 Feb 22;9:20. doi: 10.1186/s40560-021-00535-x (PMC7897892; doi:10.1186/s40560-021-00535-x)
Supplement: Supplementary file 2 — Additional file 2. Search strategy and keywords. [file 40560_2021_535_MOESM2_ESM.docx]

**Search strategy and keywords**

| PubMed | |
| --- | --- |
| 1 | coronavirus[Title/Abstract] OR COVID-19[Title/Abstract] OR SARS-CoV-2[Title/Abstract] OR 2019-nCoV[Title/Abstract] |
| 2 | (thrombus[Title/Abstract] OR thrombo*[Title/Abstract] OR embolus[Title/Abstract] OR emboli*[Title/Abstract]) |
| 3 | #1 AND #2 Filters: from 2020 - 2021 |

| Embase | |
| --- | --- |
| 1 | coronavirus:ab,ti OR 'covid 19':ab,ti OR 'sars cov 2':ab,ti OR '2019 ncov':ab,ti |
| 2 | thrombus:ab,ti OR thrombo*:ab,ti OR embolus:ab,ti OR emboli*:ab,ti |
| 3 | #1 AND #2 AND [2020-2021]/py |

| Web of Science | |
| --- | --- |
| 1 | TS=(coronavirus OR COVID-19 OR SARS-CoV-2 OR 2019-nCoV) |
| 2 | TS=(thrombus OR thrombo* OR embolus OR emboli*) |
| 3 | #1 AND #2 Timespan=Year to date |
